# Pattern change of precipitation extremes in Svalbard

**DOI:** 10.1038/s41598-025-92339-4

**Published:** 2025-03-13

**Authors:** Dhiman Das, R. Athulya, Tanujit Chakraborty, Arnob Ray, Chittaranjan Hens, Syamal K. Dana, Dibakar Ghosh, Nuncio Murukesh

**Affiliations:** 1 Physics and Applied Mathematics Unit, Indian Statistical Institute, Kolkata, 700108 India; 2 National Centre for Polar and Ocean Research, Ministry of Earth Sciences, Vasco da Gama, 403804 India; 3 Present Address: SAFIR, Sorbonne University Abu Dhabi, Abu Dhabi, United Arab Emirates; 4 Sorbonne Center for Artificial Intelligence, Sorbonne University, 75005 Paris, France; 5 Center for Computational Natural Science and Bioinformatics, International Institute of Informational Technology, Hyderabad, 500032 India; 6 Division of Dynamics, Lodz University of Technology, 90-924 Lodz, Poland; 7 Centre for Mathematical Biology and Ecology, Department of Mathematics, Jadavpur University, Kolkata, 700032 India

**Keywords:** Precipitation extremes, Arctic region, Extreme value theory, Generalized Pareto distribution, Return levels, Applied physics, Statistical physics, thermodynamics and nonlinear dynamics, Climate sciences

## Abstract

Besides global attention on extreme precipitation, a limited research has been done in the Arctic due to constraints of data availability. In this backdrop, we attempt to analyze extreme precipitation events at three Arctic stations (Bjørnøya, Ny-Ålesund, and Svalbard Lufthavn) in Svalbard using extreme value theory. The analysis revealed that these high-latitudinal Arctic stations were characterized by heavy-tailed distributions for the exceedances, suggesting a higher probability of the occurrence of extreme precipitation events. Ny-Ålesund and Bjørnøya have exhibited a significant increase in return values over the last three decades. Among the three stations, Ny-Ålesund displayed the strongest return values, especially in winter post-1994 when the atmospheric temperature was characterized by an enhanced positive trend. Significant seasonal variability in return values has also been observed; the fall in Ny-Ålesund was characterized by a low-intensity regime as indicated by the shape parameter. Ny-Ålesund precipitation had shifted from heavy-tailed distribution in pre-1994 to bounded tail distribution post-1994 during spring. Bjørnøya’s extremes are driven by cyclonic circulation, while southerly winds drive extremes in Ny-Ålesund and Svalbard Lufthavn. Even though, Svalbard Lufthavn, displayed regime changes, showed low variability, likely due to its position in a rain shadow region. This research highlights the nuanced responses of Arctic hydrology to warming, emphasizing the need for localized studies and active collaboration with policymakers to translate these insights into effective climate adaptation and mitigation strategies.

## Introduction

Global climate change intensifies the frequency and severity of precipitation extremes, posing significant challenges to ecosystems, infrastructure, and human livelihoods^1,2^. The changes are particularly pronounced in the Arctic, where warming occurs at more than twice the global average-a phenomenon known as Arctic Amplification^3,4^. This enhanced warming increases the atmosphere’s moisture-holding capacity, fueling stronger and more intense precipitation patterns^5^. A comparison of observation and multimodal simulation indicates that intensification of heavy precipitation over the Northern Hemisphere land areas is primarily driven by human-induced increase in greenhouse gas concentrations ^6,7^. Arctic precipitation is projected to rise further in response to rapid atmospheric adjustments on short time scales and slower oceanic mechanisms on longer ones ^8^. Additionally, increases in annual mean, daily, and 5-day total precipitation are strongly linked to enhanced atmospheric moisture from warming and intensified moisture transport from lower latitudes towards the Arctic ^9^. A predominantly frigid Arctic is expected to experience frequent and intense precipitation events, accompanied by a marked shift from snow to rain ^10^.

A recent work by Athulya et al.^11^ in Ny-Ålesund, in the high Arctic coastal region, highlights a significant rise in winter precipitation without any corresponding increases in the number of precipitation days. This suggests that, in a warmer Arctic, intense short-duration precipitation events may become more frequent^12,13^. This poses a question: Whether Arctic precipitation is shifting towards extremes? A shift in the precipitation pattern in the Arctic can affect the entire Arctic hydrology by melting glaciers and ice caps, affecting ocean circulation and weather patterns, and eventually contributing to sea level rise^14,15^. Another important factor affecting precipitation by the changing atmospheric circulation is the sea ice reduction. For example, the Barents Sea, a deep water-forming site in the Arctic, experienced a dramatic reduction in the sea ice^16^, significantly altering the local atmospheric circulation patterns. Given the potential influence of extreme weather events on the Arctic hydrological cycle, a comprehensive understanding of the occurrences of extremes is crucial for better projection of climate and weather in the high Arctic and their impact on the oceanic circulation.

While several studies have explored extreme precipitation patterns globally ^17–21^, research in the Arctic remains limited, constrained by data availability. Long-term precipitation trends are documented only at a few Arctic stations; the Svalbard archipelago, in the Arctic, is a crucial site due to its reliable climate records and favorable location within the area of amplified Arctic warming ^22–24^. Svalbard winters are marked by extreme precipitation driven by southwesterly advection ^25–27^. The extreme precipitation trends in Svalbard from 1979 through the early 2000s showed no consistent temporal pattern ^28^. However, a recent study in Ny-Ålesund, observed a significant increase in precipitation during fall but noted that winter extremes displayed a positive trend only between 2000-2019 ^11^. These findings contradict estimates from reanalysis datasets, underscoring the need for a more precise understanding of station-based data ^29^. A study by Forland et al.^24^ analyzed five stations in Svalbard for a hundred years (till 2011) and compared the trends in temperature and precipitation. The mean annual precipitation at Ny-Ålesund was observed to be more than twice that of Svalbard Lufthavn^24^. Additionally, it was noted that Svalbard Lufthavn and Ny-Ålesund recorded the highest daily rainfalls of 43 and 98 mm, respectively, in 2012, which constituted $25\%$ of the average annual rainfall in Svalbard^30^. Extreme events differ seasonally, interannually, and geographically, highlighting the complexity of spatio-temporal variability in precipitation across the Svalbard archipelago ^25–27^, where most of the stations are located near the ocean, making them particularly suitable for studying oceanic precipitation. Oceanic precipitation constitutes about $60\%$ of total precipitation over the Arctic land areas ^31^, representing a critical but underexplored aspect of the Arctic hydrological cycle.

In this perspective, we explore intense precipitation events or extremes at three stations in Svalbard using extreme value theory (EVT). We use the peak over threshold (POT) method for identifying precipitation extremes. Classical statistical methods primarily focus on the average behavior of stochastic processes, whereas EVT concentrates on the tail of the distribution, making it particularly relevant for studies of rare and high-impact occurrences. EVT provides a robust model and framework to describe unusual events often encountered in geoscience applications, such as extreme precipitation, heatwaves, and floods^32–38^. This statistical theory offers powerful methodologies for modeling extremes, including the generalized extreme value (GEV) distribution and the generalized Pareto distribution (GPD). These approaches enable accurate extrapolation of probabilities and magnitudes beyond observed data, such as return levels for 50 or 100 years, providing realistic risk assessments. In climate studies, infrastructure design, and disaster preparedness-where conventional methods often underestimate the hazards of catastrophic events-EVT proves indispensable. Its distribution-independent nature, ability to account for heavy tails, and flexibility to incorporate trend behavior make it an essential tool for addressing the challenges posed by extreme climatic events.

In “Datasets and methods”, we describe the location of the Svalbard archipelago, the datasets used, and the methods employed. A discussion on POT approach is included in the Sect. I of Supplementary information. In “Results”, we apply the POT method to model precipitation extremes at three stations in Svalbard. We analyze comparative studies of precipitation extremes for the two consecutive time intervals separately based on Arctic-wide temperature changes in “Changes in the pattern of precipitation extremes during a recent warming period in the Arctic”. Finally, results are summarized in “Discussion and conclusion”.

**Fig. 1:**
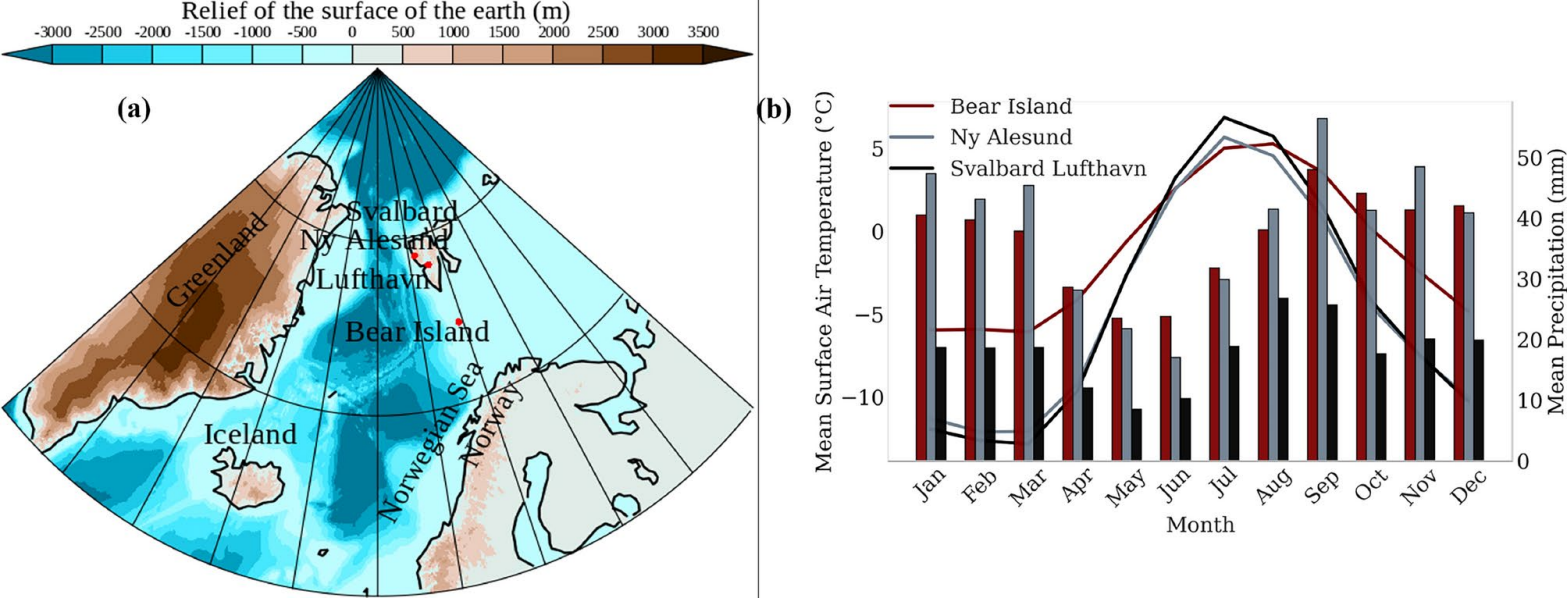
(**a**) Locations of Bjørnøya (Bear Island), Ny-Ålesund, and Svalbard Lufthavn (Lufthavn) are ($74.4522^{\circ }$ N, 19.1152$^{\circ }$ E), (78.9$^{\circ }$ N, 11.9$^{\circ }$ E), and (78.25$^{\circ }$ N, 15.49$^{\circ }$ E), respectively. The color contour indicates the altitude expressed as relief of the surface of the earth (m) obtained from etopo5 in blue, indicating negative altitude (depth), and the brown color indicates positive altitude (height). Three locations are denoted by solid red circles to illustrate the variations in their geographical positions. The altitude of the stations are 8 m, 28 m, and 18 m for Ny-Ålesund, Svalbard Lufthavn and Bjørnøya, respectively. (**b**) The climatology of daily mean surface temperature ($^{\circ }$C) (line plot) and mean precipitation (mm) (bar plot) of Bjørnøya (red), Ny-Ålesund (grey) and Svalbard Lufthavn (black).

## Datasets and methods

Daily precipitation data of the meteorological stations at Bjørnøya (No. SN99710), Ny-Ålesund (No. SN99910), and Svalbard Lufthavn (No. SN99840), managed by the Norwegian Centre for Climate Services in Svalbard, were retrieved from the database available at https://seklima.met.no/. The dataset spans multiple periods across the three Arctic locations: Bjørnøya - from January 1, 1964 to December 31, 2023, Ny-Ålesund from January 1, 1975 to December 31, 2023, and Svalbard Lufthavn from January 1, 1976 to December 31, 2023. Figure 1a shows the locations of all three stations (solid red circles). While Bjørnøya is a relatively small island, Ny-Ålesund and Svalbard Lufthavn are coastal stations situated near the sea. All three locations employ precipitation gauges to record their data. However, it is important to note that these gauges are subject to biases, particularly undercatch, which occurs during periods of strong winds. This can lead to an underestimation of actual precipitation amounts, affecting the accuracy of long-term precipitation measurements, especially in harsh Arctic conditions, where wind speeds can be significant^39^. It may be argued that the recent positive trends in precipitation in Svalbard could be due to the increase in wet snowfall and rainfall, which is more efficiently captured in the precipitation gauges compared to snowfall^40^. Figure 1b presents the climatology of daily mean surface temperature ($^{\circ }$C) (line plot) and mean precipitation (mm) (bar plot) for the three stations. Ny-Ålesund (grey) and Svalbard Lufthavn (black) show a close resemblance in temperature climatology. Bjørnøya (red) shows a higher temperature during cold months compared to the other stations. Precipitation is strong during colder months and weak during warmer months across all the stations, reflecting a seasonal variation typical in maritime Arctic climates-wetter winter and drier summer.

To mitigate the biases caused by undercatch in precipitation gauge measurements, corrections are applied to improve data accuracy. Various correction methods are employed, including adjusting for wind-induced losses, wetting loss, and evaporation loss. These factors are particularly significant in the Arctic’s cold and stormy conditions. These corrections are crucial to obtain reliable precipitation estimates, especially when analyzing long-term trends and extreme events in polar regions^40^. Here, we have employed the technique followed by Kochendorfer et al.^41^ using wind speed at 10 m ($$w_{10}$$), gauge precipitation ($$P_g$$) and gauge temperature ($$T_g)$$. The correction factor for rain is given by $$k_{\text {rain}} = \left( \exp \left( -0.0281 \cdot w_{10} \left( 1 - \arctan \left( 1.628 \cdot T_g \right) + 0.837 \right) \right) \right) ^{-1}$$ and for snow is given by $$k_{\text {snow}} = \left( 0.742\cdot e^{-0.181 \cdot w_{10}} + 0.322 \right) ^{-1}$$. The wetting loss ($$\Delta p_w$$) and evaporation loss ($$\Delta p_e$$) is calculated as 0.15 mm for rain and 0.1 mm for snow^42^ and the corrected precipitation is given by $$(P_g + \Delta p)k$$. Even though this method is designed for automatic gauges, the same method can be applied to the Norwegian gauges established in the above-mentioned three stations, since the ratio of precipitation data from Norwegian gauges and automatic gauges is close to one^40^.

In earlier studies, EVT^35–38,43–45^ was usually applied to study precipitation. It allows characterization and modeling of extreme events through the use of extreme value distributions, which represent a limiting behavior of minimum or maximum data values^33,34^. We employ the peaks over threshold (POT) approach on datasets of the corrected precipitation (described above). In the POT method, any events that exceed a specified threshold are only considered as extreme events and included in the dataset; it assumes a generalized Pareto distribution (GPD) of excesses of events over the prescribed threshold. We define the threshold as 99-th percentile of the data, as a standard approach in this field^46^ (described in Sect. II of Supplementary information). We model the excess part of precipitation extremes from the threshold with GPD and estimate the return level of precipitation extremes.

Our aim is to investigate concurrent changes in extreme precipitation patterns under the Arctic warming scenario using the POT method. By analyzing the trend of Arctic temperature, we are able to identify the transitions that may represent regime changes, e.g., from low precipitating to extreme precipitating periods. In our analysis, the year 1994 appears to be a year of transition. Based on this information, we split the whole time period into two periods, (a) before 1994 and (b) from 1994 to 2023. We also analyze and plot return levels to estimate the amount of precipitation extremes during specified return periods. These return levels describe the amount of precipitation that is expected to be equaled or exceeded once in a certain time period (e.g., 10 years, 50 years, or 100 years). A visualization of these return levels provides a probabilistic framework for understanding and measuring the intensity of rare precipitation occurrences, and thereby assisting the assessment of extreme event risks. In interpreting the results using the generalized extreme value theory (GEVT), the shape parameter of the distribution is an indication of the distinct regimes. It suffices to state here that if the shape parameter $\xi < 0$, we can expect precipitation extremes with low probability with an upper bound, while the shape parameter $\xi >0$ indicates a high probability of getting precipitation extremes. For the shape parameter $\xi =0$, the chances of occurrence of extremes decrease exponentially. For more details, refer to Sect. I in Supplementary information.

## Results

### Pattern of precipitation extremes in the three locations of Svalbard

**Fig. 2:**
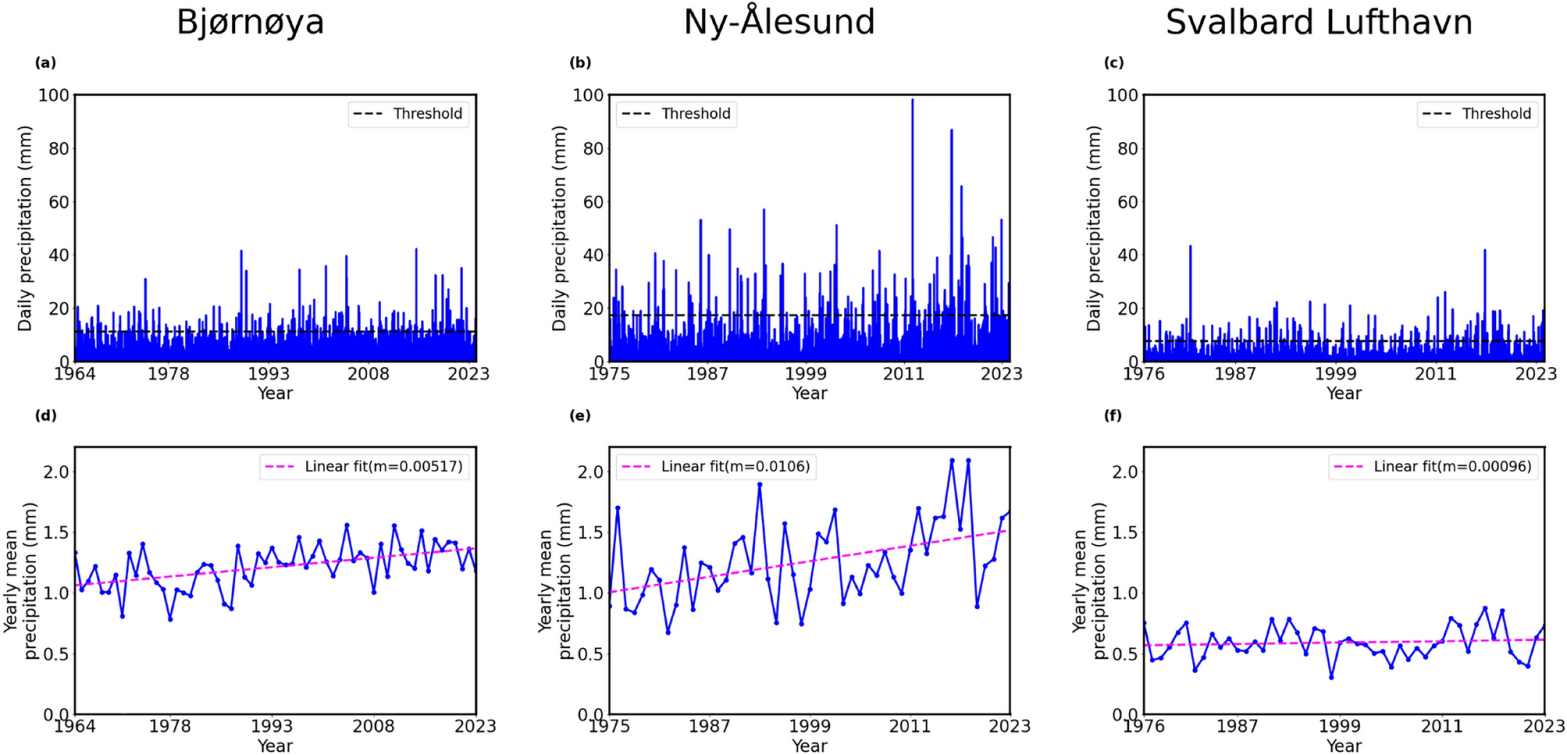
(**a**–**c**) Temporal records of daily precipitation in the three stations of Svalbard from Bjørnøya, Ny-Ålesund and Svalbard Lufthavn. The horizontal black dashed line represents 99th percentile of the dataset consisting of daily precipitation over the period, which is considered a threshold for precipitation extremes. (**d**–**f**) Variation of annual mean precipitation exhibits a linear trend fitted with a straight line (dashed magenta line).

Figure 2a–c represent temporal records of daily precipitation for the three stations in Svalbard: Bjørnøya, Ny-Ålesund, and Svalbard Lufthavn. We consider 99th percentile of the daily precipitation values as a threshold for the extreme events. Precipitation events above the threshold (horizontal black dashed lines) are considered as precipitation extremes. Ny-Ålesund exhibits strong extremes than the other two locations; Svalbard Lufthavn was characterized by smaller values for extremes. Figure 2d–f depicts the annual average of precipitation in the three stations.

**Fig. 3:**
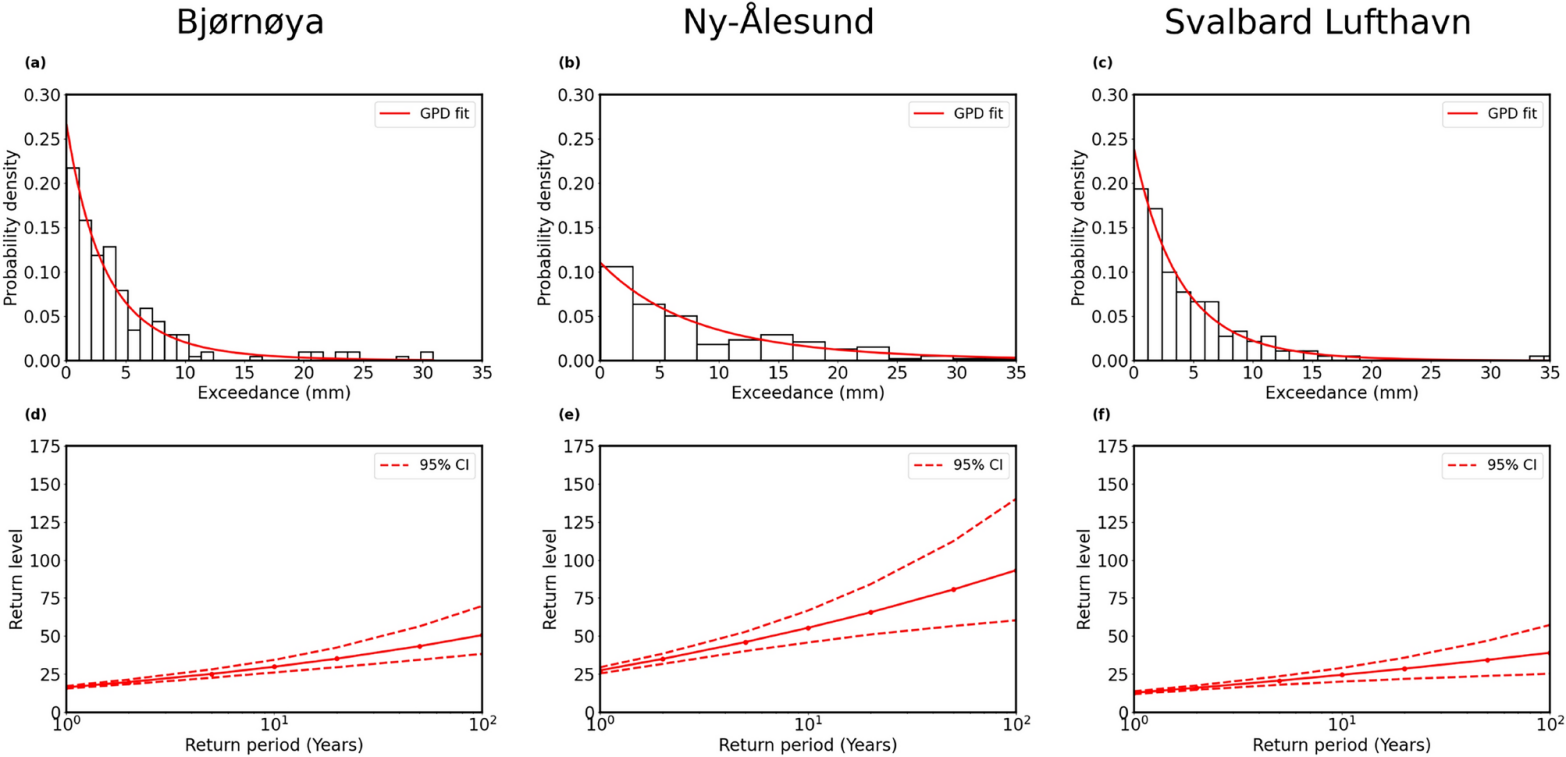
(**a**–**c**) Probability density plots of precipitation extremes are exhibited for three stations: Bjørnøya, Ny-Ålesund and Svalbard Lufthavn, respectively. All histograms are fitted with probability density (red lines) of the generalized Pareto distribution. (**d**–**f**) Return levels (red lines with solid circles) against return periods with 95% confidence interval (denoted by the red dashed lines) are shown for all the stations.

The data displayed strong interannual variability as well as linear trends. The slopes (m) of the fitted lines (dashed magenta lines) corresponding to the three specified stations were 0.00517 mm/year, 0.0106 mm/year, and 0.00096 mm/year, respectively, indicating an increasing trend of precipitation in Bjørnøya, Ny-Ålesund and Svalbard Lufthavn. The Mann–Kendall test (described in Sect. III in Supplementary information) indicated that the trend was significant for Bjørnøya and Ny-Ålesund, but not for Svalbard Lufthavn. Whenever strong interannual variability was observed in Fig. 2d–f, extreme precipitation events were also recorded, that indicating a significant contribution of extreme events to total precipitation. In fact, the analysis of annual mean total precipitation and the number of extreme precipitation events per year revealed a consistent positive trend across all the three stations (see Fig. S1 in Supplementary information). In particular, the slopes of the time series for both the variables were positive, suggesting that total annual precipitation and frequency of extreme events covary. The correlation coefficient between the annual mean and the number of extreme precipitation events exceeded 0.5 at all three locations, which indicated a moderate to strong positive correlation between the total precipitation and the occurrence of extreme precipitation events. Therefore, EVT is ideal for a limiting distribution of the extrema of precipitation observed in Svalbard for the analysis of precipitation events.

**Table 1:** Analysis of generalized Pareto distribution for precipitation extremes of Bjørnøya, Ny-Ålesund, and Svalbard Lufthavan.

Parameter estimation of the GP fitting and return level estimation						
Station (station number)	Location $(\mu )$	Scale $(\sigma )$	Shape $(\xi )$	*p*-value for KS-test	10-year return level	100-year return level
Bjørnøya (SN99710)	0.0499	3.7677	0.186	0.8531	29.9	50.7
Ny-Ålesund (SN99910)	0.0479	9.071	0.1311	0.7738	55.5	93.3
Svalbard Lufthavan (SN99840)	0.0001	4.1750	0.08928	0.8738	24.6	39.1

Using the Durbin Watson test (see Sect. IV of Supplementary information), we have first verified that the precipitation extremes do not exhibit serial correlation, indicating that the present value does not depend on past lagged observations, and then analyze the precipitation extremes using the POT approach. In this case, we fitted the histogram of the precipitation exceedances (the difference between precipitation events that exceed the pre-defined percentile-based threshold and the threshold) with the probability density function using the GPD. Three histograms of precipitation exceedance with fitted probability density function using the GPD for three stations are presented in Fig. 3a–c. The return level plots of precipitation extremes against their return periods are presented in Fig. 3d–f for the three stations, which were used to calculate the probability of precipitation extremes that exceed a defined threshold. The red lines with solid circles showed the return level based on observation and the red dashed lines represent the $95\%$ confidence interval of the return levels. Table 1 presents the estimated location, scale, and shape parameters as obtained by statistical fitting of the generalized Pareto distribution shown in Fig. 3a–c for the recorded datasets of three stations. The estimations of return level for 10-year, and 100-year return periods are also presented in the same table. Kolmogorov–Smirnov (KS) test^47^ (see Sect. V of Supplementary information) is a process of goodness-of-fit that is performed on the dataset. The *p*-values given in Table 1 indicate that the empirical distribution is appropriately fitted with the GP distribution. The positive values of the shape parameters of the GPD corresponding to precipitation extremes (exceedances) of the three stations indicate that the three distributions follow a heavy (fat) tail. It indicates that the probability distributions of extreme precipitation in all the stations follow the Fréchet family of distribution suggesting that the distributions are characterized by a high probability of extreme precipitation events. The heavy tails indicate that higher magnitude precipitation extremes were more frequent, indicating a higher probability of such rare but impactful events. Furthermore, the 100-year return level values for the three stations indicated that the magnitude of extreme precipitation events occurring once every 100 years in Ny-Ålesund was the highest, suggesting greater chances of occurrence of severe precipitation events there.

**Fig. 4:**
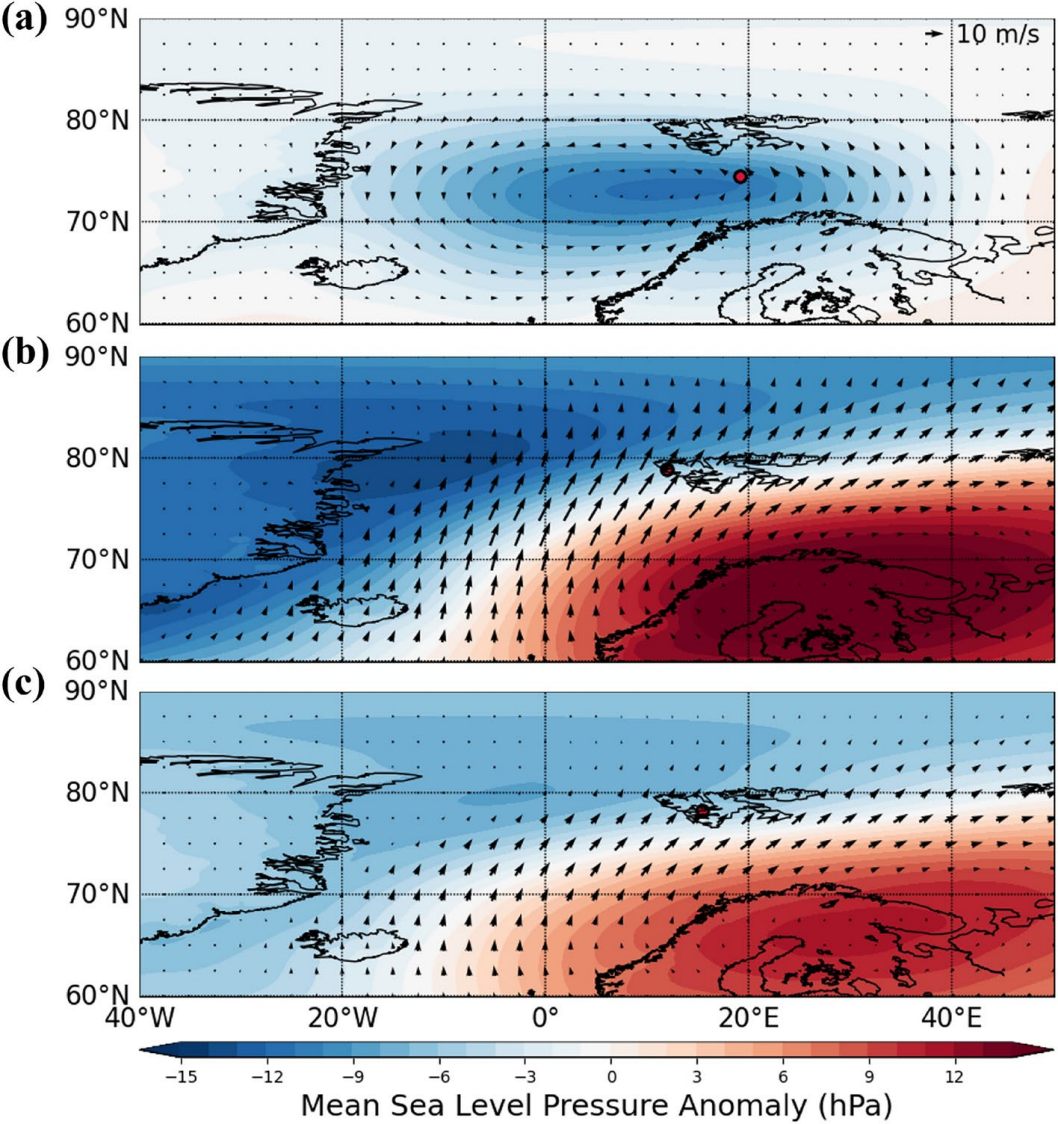
Composites of large-scale atmospheric circulation patterns during days with extreme precipitation, derived from ERA5 reanalysis, for (**a**) Bjørnøya, (**b**) Ny-Ålesund, and (**c**) Svalbard Lufthavn. The color contours represent mean sea-level pressure anomalies (hPa), with red indicating regions of higher pressure, notably over northern Europe in (**b**) and (**c**), and blue indicating regions of lower pressure. Overlaid vectors illustrate wind anomalies averaged from the surface to 750 hPa. Red circles denote three stations.

In order to understand the atmospheric characteristics, we have analyzed the sea level pressure and winds during the extreme events in Fig. 4. For extremes in Ny-Ålesund and Svalbard Lufthavn, a strong high-pressure anomaly is noticed over North/Northwestern Europe, which could be a result of a reduction of sea ice^48^. This pattern is expected to transport heat and moisture into Svalbard^49,50^. In recent years, the high-pressure anomaly displayed an increase in intensity^51^ and could be forced by remote mechanisms as well^52^. The high-pressure anomalies could be a reason for extremes in Svalbard over the last two millennia^22^. However, similar circulation patterns did not result in similar precipitation characteristics for Svalbard Lufthavn and Ny-Ålesund. Currently, we do not have a strong explanation for this dichotomy. One reason could be that intense precipitation occurs along with the atmospheric rivers, which depict high spatio-temporal variability. It may also be due to local factors, viz., topography or the atmosphere/oceanic temperature gradients that play a role in shaping precipitation extremes in these locations. This warrants a separate investigation. Nevertheless, the sea-level pressure and circulation patterns during extreme events in Bjørnøya differed from those at other stations, with a low-pressure system centered over Bjørnøya being responsible for the extreme precipitation.

### Seasonal precipitation and circulation at each station

**Fig. 5:**
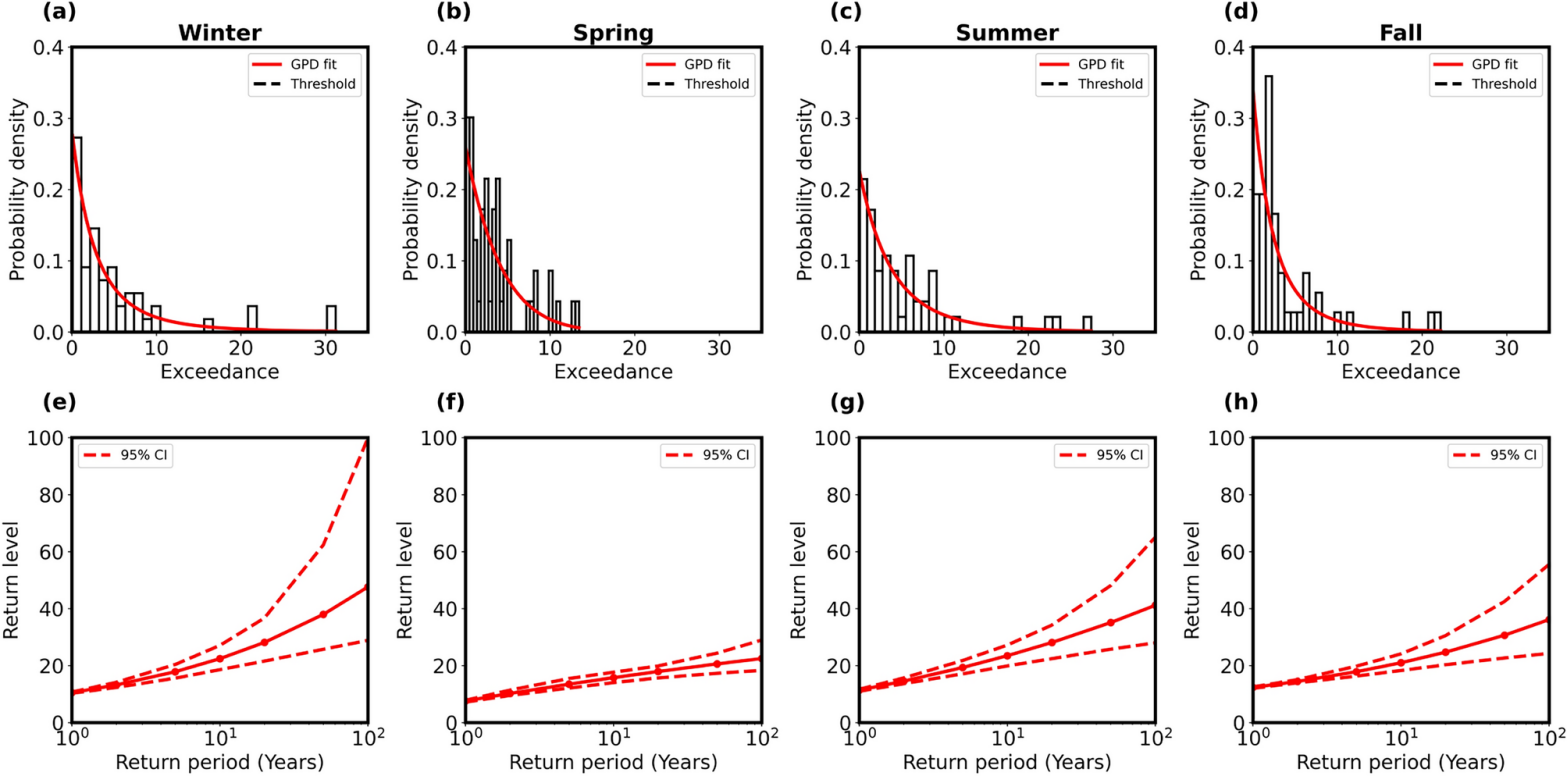
Bjørnøya station: (**a**–**d**) Histograms (black) of seasonal data of precipitation extremes fitted with probability densities (red) of GP distributions for four seasons, i.e., winter, spring, summer, and fall, respectively. (**e**–**h**) Return level plots (red solid lines) from statistical models against return periods within $95\%$ confidence interval (red dashed lines).

**Fig. 6:**
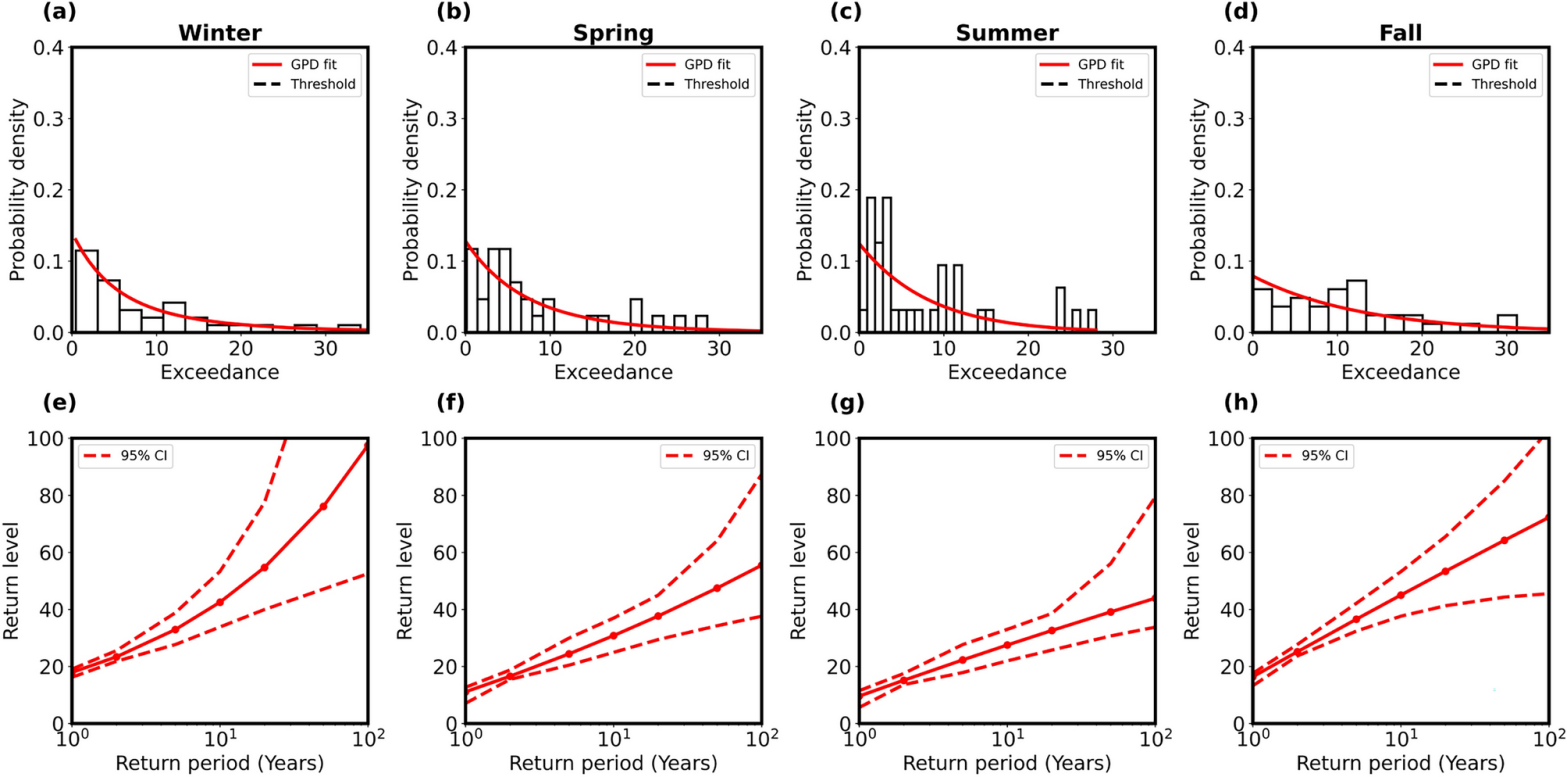
Ny-Ålesund station: (**a**–**d**) Histograms (black) of seasonal data of precipitation extremes fitted with probability densities (red) of GP distributions for four seasons, i.e., winter, spring, summer, and fall, respectively. (**e**-**h**) Return level plots (red solid lines) from statistical models against return periods within $95\%$ confidence interval (red dashed lines).

**Fig. 7:**
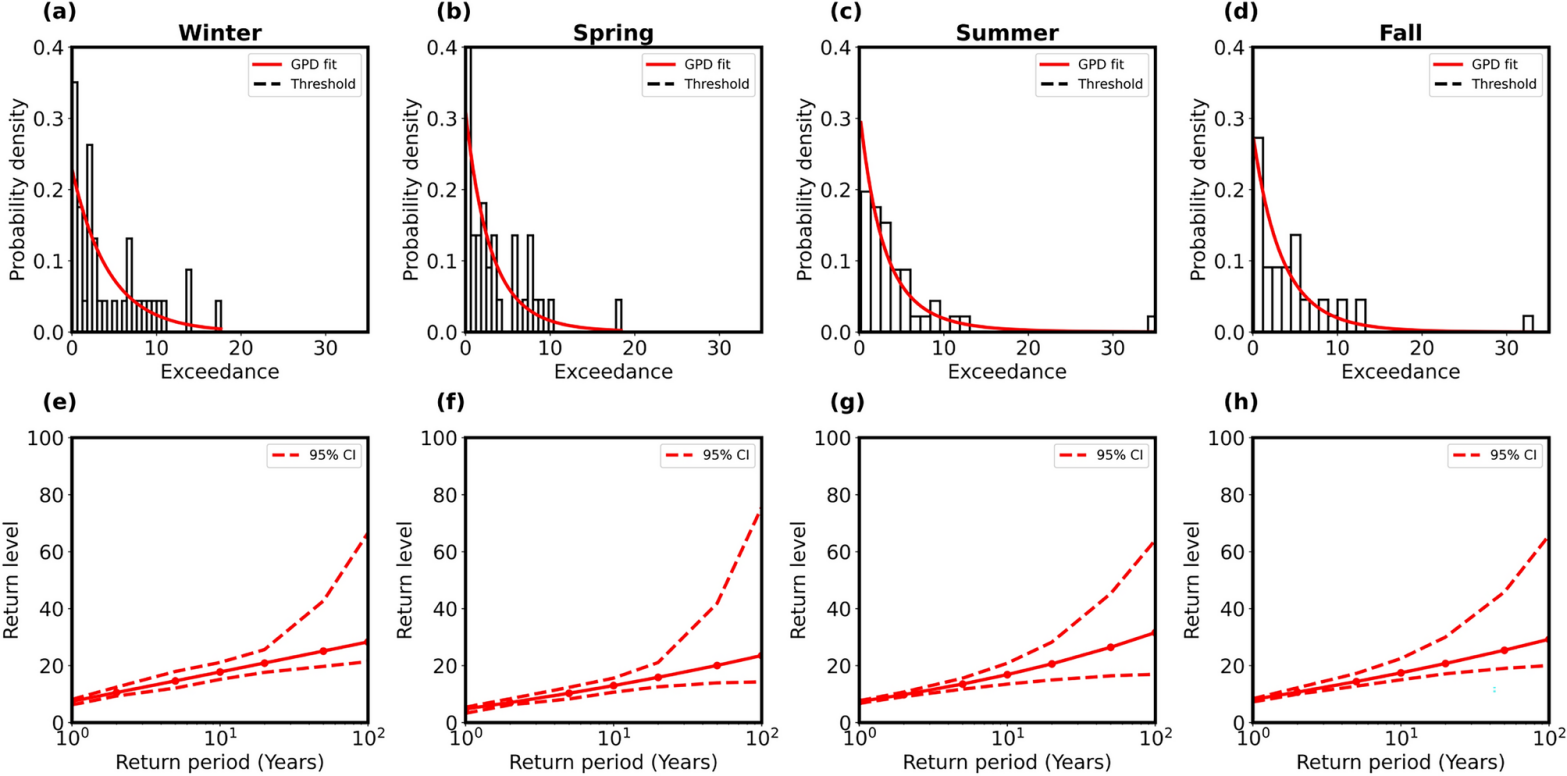
Svalbard Lufthavn station: (**a**–**d**) Histograms (black) of seasonal data of precipitation extremes fitted with probability densities (red) of GP distributions for four seasons, i.e., winter, spring, summer, and fall, respectively. (**e**–**h**) Return level plots (red solid lines) from statistical models against return periods within $95\%$ confidence interval (red dashed lines).

To further ascertain the extremes, the time series data of the three stations were segregated seasonally, and similar analyses as made above have been conducted for each season separately. The seasonal variation was studied by considering the four seasons as follows: (i) winter (December–January–February), (ii) spring (March–April–May), (iii) summer (June–July–August), and (iv) fall (September–October–November). In order to derive any statistical model of the precipitation extremes, we applied the GPD once again for each of the four seasons separately. Figures 5, 6 and 7a–d show the empirical probability density plots for the three stations for four seasons fitted with PDF (red line) of the GPD model. The return levels (red line with solid circles) as derived (using Eq. S2 in the Supplementary information) are plotted against the return periods with $95\%$ confidence levels (red dashed lines) as shown in Figs. 5, 6 and 7e–h for four seasons of the three stations. We also estimated the shape parameters for 100-year return level in Table 2 for four seasons of the three stations.

Results in Figs. 5, 6 and 7 suggest that extremes were frequent in Ny-Ålesund during winter. The 100-year return values were also maximum in this location. The shape parameter was weakly negative during summer and fall in Ny-Ålesund and during spring in Bjørnøya, indicating an upper bound to the precipitation values and a tendency for fewer extremes during the time period examined (Table 2). Svalbard Lufthavn did not show any dramatic seasonal changes, while the variability at Bjørnøya was lower than Ny-Ålesund.

**Table 2:** Shape parameter and 100-year return level for Bjørnøya, Ny-Ålesund, and Svalbard Lufthavn for four seasons (winter, spring, summer and fall) are estimated from GPD model fitting in Figs. 5, 6 and 7a–d.

Season	Bjørnøya		Ny-Ålesund		Svalbard Lufthavn	
	Shape $(\xi )$	100-year RL	Shape $(\xi )$	100-year RL	Shape $(\xi )$	100-year RL
Winter	0.32512	47.6	0.34968	97.5	0.01079	28.3
Spring	− 0.08566	22.5	0.09761	55.5	0.10953	23.5
Summer	0.16615	41.2	− 0.03834	43.9	0.19253	31.6
Fall	0.24104	36.2	− 0.02161	72.3	0.09840	29.2

**Fig. 8:**
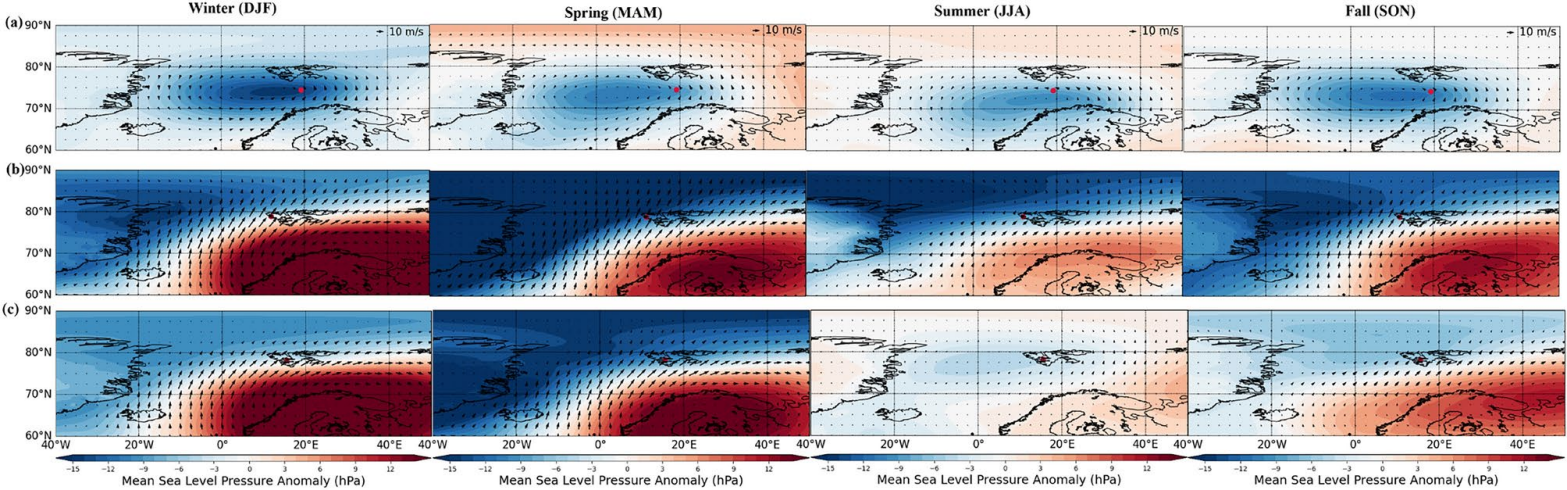
Composites of large-scale atmospheric circulation patterns during the days with extreme precipitation obtained from ERA5 reanalysis in (**a**) Bjørnøya, (**b**) Ny-Ålesund, and (**c**) Svalbard Lufthavn. The color contours show mean sea-level pressure anomaly (hPa), and the vectors are the wind anomalies averaged from the surface to 750 hPa.

### Changes in the pattern of precipitation extremes during a recent warming period in the Arctic

Figure 8 illustrates the seasonal (four seasons separately) atmospheric circulation patterns during extreme precipitation at each station. Overall, the seasonal circulation patterns at each station did not exhibit significant variations apart from changes in intensity, thus resembling the annual circulation patterns. Generally, the cold seasons (winter and fall) displayed stronger circulation, while summer exhibited weaker circulation. Both Ny-Ålesund and Svalbard Lufthavn demonstrated similar patterns during extreme precipitation events despite the difference in the dates of the events at each station. Similar to the annual circulation patterns, high pressure over northern Europe and low pressure over the Arctic with southwesterly wind advection was observed during the extremes in Ny-Ålesund and Svalbard Lufthavn. A cyclonic circulation was observed in Bjørnøya.

**Fig. 9:**
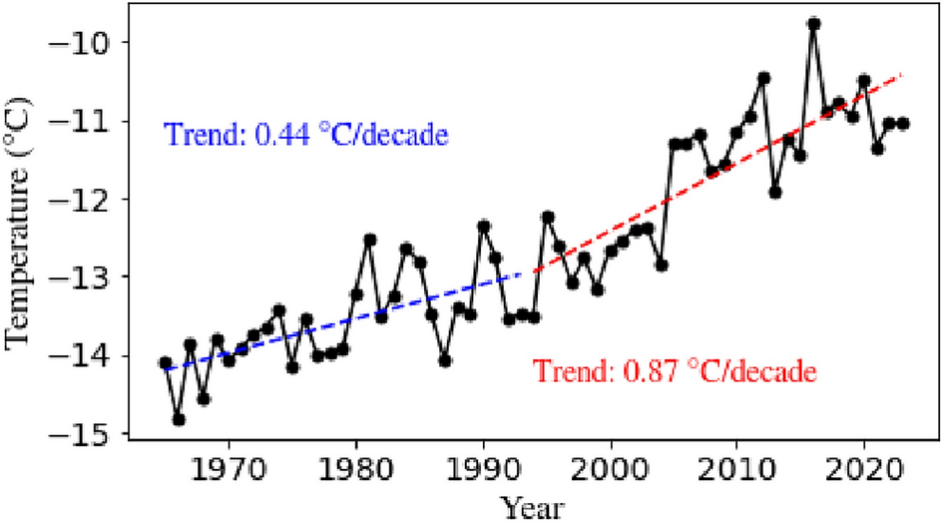
Trend in annual surface temperature at 2 m averaged over the Arctic (72$^{\circ }$ N–90$^{\circ }$ N and 0$^{\circ }$ E–360$^{\circ }$ E) from 1965 to 2023 obtained from ERA5 reanalysis. The trend is calculated separately for two periods, 1965–1993 and 1994–2023, as significant warming has been observed in the data since 1994. The significance of both trends is calculated using the Mann–Kendall test (described in Sect. III of Supplementary information), and the trends for both periods are found to be significantly increasing.

Next, we examine the changes in the occurrence of precipitation extremes during the recent Arctic warming period. The Arctic is undergoing rapid warming, a phenomenon known as Arctic amplification. As discussed earlier, precipitation is expected to increase in a warmer atmosphere due to enhanced evaporation from open water^10^. Notably, the stations considered in this study are located within the region experiencing accelerated warming in the Arctic (see Fig. 1 of Ref.^53^). Our approach aims to understand the impacts of Arctic-wide warming at individual locations. These changes are driven by factors such as increased greenhouse gas concentrations, large-scale circulation shifts, and sea-ice reduction. Analysis of the annual average air temperature time series, derived from ERA5 reanalysis data^54^ and averaged over the 70$^{\circ }$ N–90$^{\circ }$ N, revealed two distinct periods. Since 1994, temperatures have risen significantly, exhibiting a steeper trend compared to the period before 1994, as shown in Fig. 9.

The air temperature trend for the period 1994–2023 was nearly double that of 1965–1993, indicating an accelerated warming rate in recent decades. We have also examined the individual station records to ascertain whether the Arctic-wide changes are reflected in the station data (Fig. S2 in Sect. VII of the Supplementary information). In all three stations, we identify the accelerated shift that occurred during the mid to late 1990s, reflecting the large-scale changes in the Arctic. It is pertinent to note here that Svalbard Lufthavn warmed at a faster rate than the rest of the two stations. In the later half, Svalbard Lufthavn and Ny-Ålesund depicted similar trends. The covariation between precipitation extremes and temperature shifts in Svalbard was evident across Ny-Ålesund and Bjørnøya, but Svalbard Lufthavn stood out for its lack of significant precipitation shifts during the discussed period.

**Fig. 10:**
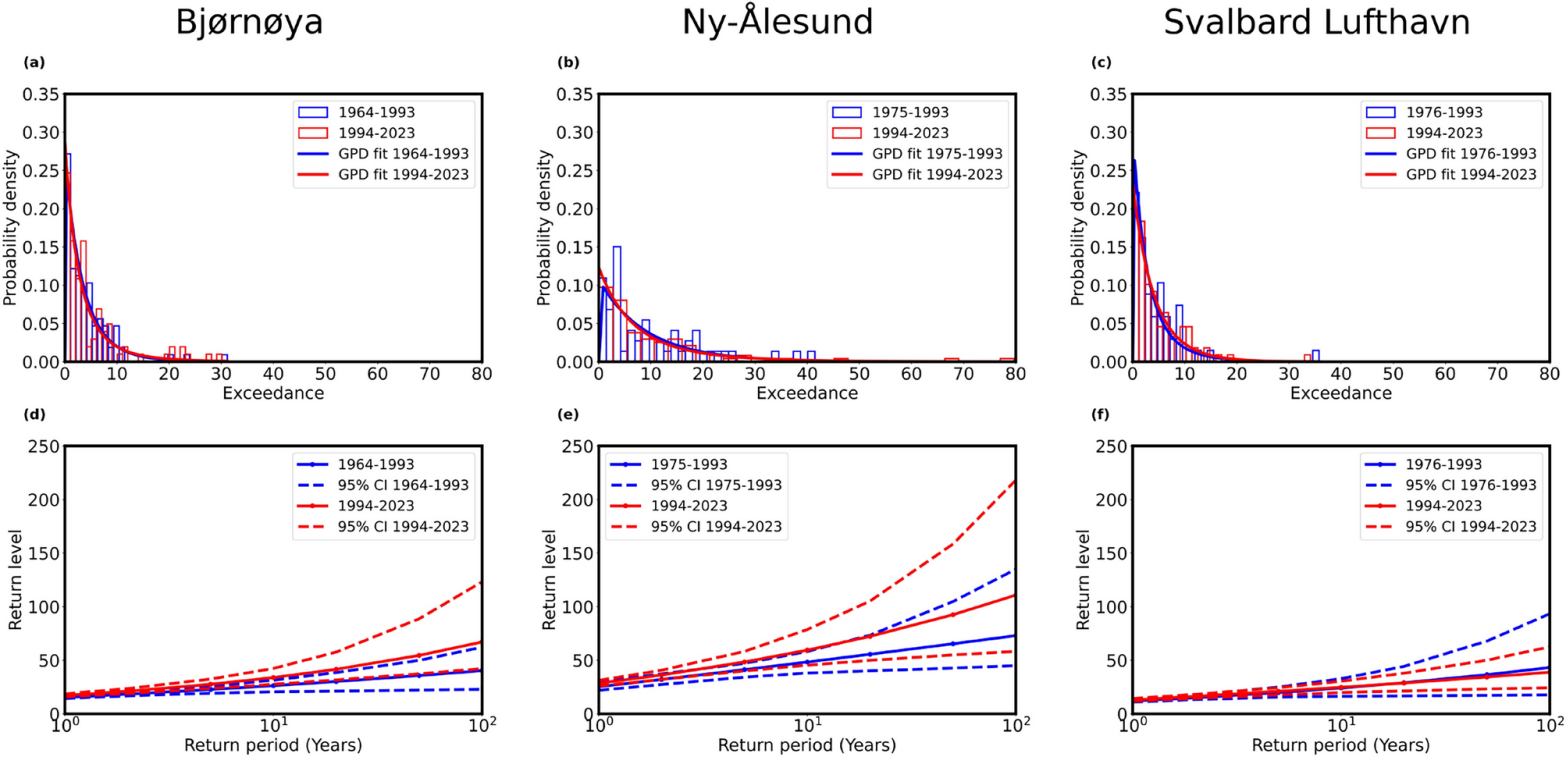
(**a**–**c**) Histograms of exceedances and the estimated probability density function of generalized Pareto distribution are illustrated for two consecutive time spans. (**d**–**f**) We plot the return level (solid lines) for different return periods with $95\%$ confidence intervals (dashed lines). Before 1994, all the plots are shown in blue, whereas from 1994 to 2023, all results are depicted in red.

Hence, we split the time period associated with each station into two intervals based on Arctic-wide temperature changes: One is from 1964 up to 1993, and the other from 1994 to 2023. We performed a comparative analysis of the frequency of exceedance of precipitation extremes for the two successive time intervals separately and present the probability density functions using the GPD model in Fig. 10a–c for three respective stations: Bjørnøya, Ny-Ålesund, and Svalbard Lufthavn. Also, the return levels of the two subsequent time intervals are plotted against the return period with bounds of $95\%$ confidence interval (shown by dotted lines) in Fig. 10d–f. The shape parameters and 100-year return level are estimated for the datasets of three stations and two time periods in Table 3. It is evident that the values of the 100-year return level (approximated values: 40.2, 72.98) for the period before 1994 were less than the values of the 100-year return level (approximated values: 67.05, 110.81) for the period 1994–2023 for Bjørnøya and Ny-Ålesund. However, the return level decreased for Svalbard Lufthavn, indicating a reduction in extremes. It may also be noted that the change in the shape parameter was more than an order of magnitude for Ny-Ålesund and twice Bjørnøya, suggesting strong shifts between the epochs, except for Svalbard Lufthavn, where the shape parameter decreased. The shape parameter for Ny-Ålesund was close to zero before 1994, suggesting that the probability for high exceedances decreased exponentially, while after 1993, the probability of exceedances remained high. While, in Svalbard Lufthavn, the probability of exceedances reduced. Thus, even though a stronger temperature trend was noticed in Svalbard Lufthavn, precipitation has not responded to the warming. We also carried out the analysis based on the time shifts at individual stations (in Fig. S2 in the Supplementary information), however, no significant changes in the extreme value statistics were noticed (shown in Table S1 and Fig. S3 in the Supplementary information).

The issue is an expected amount of precipitation for extremes in the future, based on a comparison of pre-and post-climate change data. The curve of the return level for the 1994–2023 (indicated by the red line) dominated over the first time interval (illustrated blue line) for Bjørnøya and Ny-Ålesund, as shown in Fig. 10d–f, indicating a clear upward tendency in precipitation extremes in the future. Furthermore, the rate of increase in extremes during the past 30 years has been higher compared to the preceding period (before 1994).

**Table 3:** Shape parameters and 100-year return levels (RL) for Bjørnøya, Ny-Ålesund, and Svalbard Lufthavn for two consecutive time periods.

Time period	Bjørnøya		Ny-Ålesund		Svalbard Lufthavn	
	Shape $(\xi )$	100-year RL	Shape $(\xi )$	100-year RL	Shape $(\xi )$	100-year RL
Before 1994	0.10290	40.3	0.02866	73.0	0.19440	43.2
1994–2023	0.29893	67.1	0.21949	110.8	0.07488	38.8

**Table 4:** Shape parameters and 100-year return levels (RL) for Bjørnøya, Ny-Ålesund, and Svalbard Lufthavn for four seasons of two consecutive time periods before 1994 and 1994–2023.

Season	Bjørnøya		Ny-Ålesund		Svalbard Lufthavn	
	Shape $(\xi )$	100-year RL	Shape $(\xi )$	100-year RL	Shape $(\xi )$	100-year RL
Before 1994						
Winter	0.41539	41.7	− 1.17027	32.1	0.13047	26.9
Spring	0.63551	36.6	0.63861	102.1	− 1.22335	16.1
Summer	− 0.08454	30.0	0.07110	52.1	0.26949	47.3
Fall	0.11471	31.1	− 0.33627	50.8	− 0.22953	14.0
1994–2023						
Winter	0.25133	50.2	0.28749	112.8	− 0.24871	26.3
Spring	0.60989	47.3	− 0.20239	43.6	0.43160	27.8
Summer	0.28361	52.8	0.04325	41.0	− 0.13318	19.3
Fall	0.42695	44.6	0.05860	79.7	0.12339	39.2

We also attempt to address the seasonal changes that occur during the two epochs considered earlier (Table 4). We noticed significant variability in the distribution of extreme events across the three stations. In Bjørnøya, all seasons were marked by an increase in return level post-1994, however, one distinction was the change in shape parameter to positive during summer post-1994, indicating a regime change during summer in Bjørnøya. Conspicuous changes were also recorded in the other two stations. A high return value and strong positive shape parameters were noticed during spring in Ny-Ålesund before 1994. However, the shape parameter turned negative post-1994, indicating lower extremes. Thus, spring in Ny-Ålesund shifted from an extreme event-dominated regime to a non-extreme one post-1994. Exactly opposite regime changes were noticed for fall and winter. Post-1994, both seasons turned to regimes characterized by extremes. Even though the return levels were weak, Svalbard Lufthavn also underwent regime changes from extremes in summer and winter before 1994 to calmer precipitation events since 1994. Opposite characteristics were noticed for fall and spring. Across the seasons, the atmospheric temperature also showed an increase. A question arises: Does precipitation show any co-variability with temperature observed in different seasons? From the three stations studied, Bjørnøya was characterized by increased return levels during the time period 1994–2023 in all the seasons. Svalbard Lufthavn and Ny-Ålesund behaved differently. For example, in Ny-Ålesund precipitation during spring was characterized by a reduction in extremes, while the fall and winter extremes have increased. Even though summer was characterized by an accelerated trend since 1994, precipitation extremes did not increase, suggesting the role of non-extreme precipitation contributing to precipitation trend since 1994 in summer. Non-extremes are defined as precipitation lower than the threshold.

Do these changes coincide with the Arctic-wide temperature changes? Annual averages suggest an increase in extreme events in all the stations as the temperature increases, however, seasonally there are differences. Bjørnøya is the only station where return values have increased, and shape parameters have turned positive in all seasons. In Ny-Ålesund, winter and fall were characterized by more extremes. In Lufthavn, the return values were generally low, however, extremes have increased in spring and fall.

## Discussion and conclusion

The present study describes precipitation in Svalbard with a focus on understanding the occurrence, variability, and characteristics of precipitation extremes in the context of Arctic warming using the extreme value theory. We have employed the peak over threshold method to identify and characterize the precipitation extremes. By modeling precipitation exceedances above a predefined threshold with a generalized Pareto distribution, we have estimated the key parameter values that classify precipitation extremes and assess their behavior, whether they follow a heavy-tail distribution or not. The analysis has enabled us to compute the return levels against the return periods, offering valuable insights on the likelihood of future extreme precipitation events. For a better comparison of the behavior of extremes, after identifying the period of amplified warming (after 1994), the study period has been partitioned into two distinct intervals: before 1994 and from 1994 to 2023. The results indicated that the response of precipitation extremes to Arctic warming was most pronounced at Ny-Ålesund, followed by Bjørnøya and Svalbard Lufthavn, with notable seasonal variations in all stations. It should be noted that the precipitation amount also had a similar pattern with the highest at Ny-Ålesund followed by Bjørnøya and Svalbard Lufthavn. While Ny-Ålesund exhibited the greatest variability, it experienced regime shift in all seasons except summer during the accelerated warming period. Significant alterations, characterized by large return values, were observed during spring (before 1994) and winter (after 1994) in Ny-Ålesund. Specifically, spring in Ny-Ålesund was associated with stronger extremes/weaker extremes before/after 1994, whereas winter displayed an opposite trend. In Bjørnøya, however, it was the summer when we observed higher return values after 1994. In contrast, Svalbard Lufthavn demonstrated regime changes in all the seasons. Fall and spring were characterized by more moderate conditions before 1994, transitioning to regimes marked by extremes after this year. Summer and Winter are defined by extreme/non-extreme conditions pre/post-1994. It is interesting to note that Ny-Ålesund has a negative shape parameter during summer and fall, suggesting low-impact events (Table 2).

The atmospheric circulation during precipitation extremes in the Arctic revealed contrasts between locations. High-pressure systems over northwestern Europe, paired with southerly to southwesterly winds, dominate Ny-Ålesund and Svalbard Lufthavn precipitation. These winds drive large-scale moisture transport into the Arctic, with Ny-Ålesund particularly affected in winter, as reflected by enhanced return levels and shape parameters. This is supported by studies showing that moisture fluxes linked to these wind patterns enhance precipitation extremes, as seen in various Arctic locations^11,55,56^. In contrast, Svalbard Lufthavn, experiencing the same circulation pattern, showed dampened precipitation intensity as it has a lesser maritime climate compared to the other two stations^57^ and its position in a rain shadow region^39^. Thus, despite a warming trend noticed in Svalbard Lufthavn, extremes in precipitation do not seem to covary with it, suggesting a role of interannual and location-specific variability in driving precipitation extremes. In Bjørnøya, the circulation was cyclonic, with easterly winds over the location when extremes occurred Fig. 4a–c. Moreover, the decline in sea ice cover has been shown to disrupt typical atmospheric patterns, enhancing moisture transport and potentially altering precipitation in Svalbard, as the loss of sea ice leads to increased heat absorption by the ocean and shifts in regional atmospheric dynamics^58,59^. In Ny-Ålesund, enhanced precipitation variability reflects the interaction of moisture transport and localized topography. Meanwhile, Bjørnøya’s distinct cyclonic influences and Svalbard Lufthavn’s rain shadow effects illustrate the Arctic’s heterogeneity in response to warming. These findings underscore the need for continued, localized studies to unravel the Arctic’s evolving hydrological cycle.

## Supplementary Information

Supplementary Information.

## Data Availability

In situ data is collected from https://seklima.met.no and no new data were created for this study. For studying the atmospheric pattern, data (surface and multiple pressure levels) is downloaded from https://cds.climate.copernicus.eu/cdsappdataset.
