# Supplementary material for: Pattern change of precipitation extremes in Svalbard
Source: Sci Rep. 2025 Mar 13;15:8754. doi: 10.1038/s41598-025-92339-4 (PMC11906635; doi:10.1038/s41598-025-92339-4)
Supplement: Supplementary file 1 — Supplementary Information. [file 41598_2025_92339_MOESM1_ESM.pdf]

Supplementary information:
Pattern change of precipitation extremes in Svalbard

Dhiman Das,[1,*] R. Athulya,[2,*] Tanujit Chakraborty,[3,4] Arnob Ray,[1]
Chittaranjan Hens,[5] Syamal K. Dana,[6] Dibakar Ghosh,[1] and Nuncio Murukesh[2]

[1] *Physics and Applied Mathematics Unit, Indian Statistical Institute, 203 B. T. Road, Kolkata 700108, India*

[2] *National Centre for Polar and Ocean Research, Ministry of Earth Sciences, Vasco da Gama, 403804, India*

[3] *Department of Science and Engineering, Sorbonne University, Abu Dhabi, United Arab Emirates*

[4] *Sorbonne Center for Artificial Intelligence, Sorbonne University, Paris, 75005, France*

[5] *Center for Computational Natural Science and Bioinformatics, International Institute of Informational Technology, Hyderabad, 500032, India*

[6] *Division of Dynamics, Technical University of Lodz, 90-924 Lodz, postcode, Poland*

I Peak over threshold Method

There are two methods to find the extremes from a data series, namely block-maxima and peak over threshold methods [S1]. For our data, we use the peak over threshold (POT) approach to define the extremes [S4]. This method involves by choosing a threshold value and then identifying the extremes that exceed the threshold.

This method provides an accurate approach for estimating tail parameters by focusing on extreme values that surpass a predetermined threshold. The POT method assumes that extreme occurrences occur independently and follow a generalized Pareto distribution (GPD) over a specific threshold. By choosing an appropriate threshold, the method allows us to focus on extreme values, resulting in more accurate estimates of tail parameters than traditional methods that evaluate the entire distribution. This method models the distribution of the exceedences over the threshold (u). Random variables are $\{Y_j\ (= X_j - u) | X_j > u; j = 1, 2, ..., n\}$. The distribution of Y_j converges to GPD as $n \longrightarrow \infty$.

$$H(y; \sigma_u, \xi) = \begin{cases} 1 - \left(1 + \xi \dfrac{y}{\sigma_u}\right)^{-\frac{1}{\xi}}, & \xi \neq 0, \\ 1 - e^{-\dfrac{y}{\sigma_u}}, & \xi = 0, \end{cases} \tag{S1}$$

where $\sigma_u = \sigma + \xi(u - \mu)$.

The T year return level z_T represents the value expected to be exceeded once every T years. It is derived as

$$z_T = \begin{cases} u + \dfrac{\sigma}{\xi}\left[(\lambda T)^{\xi} - 1\right], & \xi \neq 0, \\ u + \sigma \log(\lambda T), & \xi = 0. \end{cases} \tag{S2}$$

where u denotes the threshold and λ represents the rate of exceedances. The shape parameter is given by ξ whereas σ indicates the scale parameter. The GPD has three types of domains of attraction depending on the sign of ξ. When $\xi > 0$, it represents the Fréchet family of distributions, which are heavy-tailed. $\xi = 0$ suggests the Gumbel distribution, which is light-tailed. $\xi < 0$ denotes the Weibull family of distributions known as the bounded distribution.

---

* the authors have equal contribution in this work

II Method of threshold selection: Percentile based approach

We use 99-th percentile of the dataset of precipitation for selecting a threshold of extreme events. To calculate the 99-th percentile of a dataset, start by arranging the values in ascending order, i.e., from smallest to largest. Next, we determine the position (say, P) of the 99th percentile by using the formula $P = 0.99 \times (N + 1)$, where N is the total number of data points. This calculation gives the approximate position within the sorted list where the 99th percentile lies. If this position is an integer, the 99th percentile is simply the value at that position in the ordered data. If the position is not an integer, you can find the 99th percentile through interpolation: take the two closest values around that position and estimate the percentile by calculating a weighted average between them. This final value represents the cutoff below which 99% of the data falls.

III Mann-Kendall test

The Mann-Kendall (MK) trend test is a widely used non-parametric method for detecting trends in time series data without assuming any specific data distribution [S2]. In this test, the null hypothesis (H_0) states that there is no trend in the data, while the alternative hypothesis (H_1) suggests the presence of a trend. The test calculates a statistic by analyzing differences between data points, providing an indication of whether the trend is positive or negative. If the computed p-value is below a predefined significance level α (often set at 0.05), the null hypothesis is rejected, which indicates that a statistically significant trend is present in the data. For instance, setting $\alpha = 0.05$ implies that there is less than a 5% probability that the observed trend could be due to chance alone.

IV Durbin-Watson Test

The Durbin-Watson (DW) test is used in regression analysis, particularly with time series data, to detect autocorrelation in the residuals (errors). Autocorrelation occurs when residuals are not independent, meaning that one error term may be correlated with previous error terms. In the DW test, the null hypothesis (H_0) assumes no autocorrelation, indicating that the residuals are independent. The test calculates a p-value that measures the strength of evidence against the null hypothesis. If the p-value is high (greater than 0.05), there is not enough evidence to reject the null hypothesis, suggesting no significant autocorrelation in the residuals. Conversely, a low p-value (typically below 0.05) leads to rejecting the null hypothesis, which implies that autocorrelation is present in the residuals.

V Kolmogorov-Smirvov goodness-of-fit test

The Kolmogorov-Smirnov (KS) test is a non-parametric test used to determine whether a sample comes from a specified distribution [S3]. In this test, the null hypothesis (H_0) assumes that the data follows the given distribution, while the alternative hypothesis (H_1) suggests that it does not. The KS test compares the empirical distribution function of the sample with the cumulative distribution function of the specified distribution, measuring the largest difference between them. If the p-value resulting from the test is greater than the chosen significance level (typically $\alpha = 0.05$), we fail to reject the null hypothesis, indicating that there is not enough evidence to conclude that the sample differs from the specified distribution.

VI Correlation between yearly mean precipitation and occurrence of extreme precipitation

Now we study whether there is any correlation between the yearly mean precipitation and count of extreme precipitation in each stations. We use Pearson correlation to check this. We calculate the slopes of the time series for both the variables and the slopes are positive which indicates that the total annual precipitation and frequency of extreme events are covarying (in Fig. S1). In our case, the correlation coefficient between the two time series (annual mean precipitation and the number of extreme precipitation events) exceeded 0.5 at the three stations, which indicates a moderate to strong positive correlation between them.

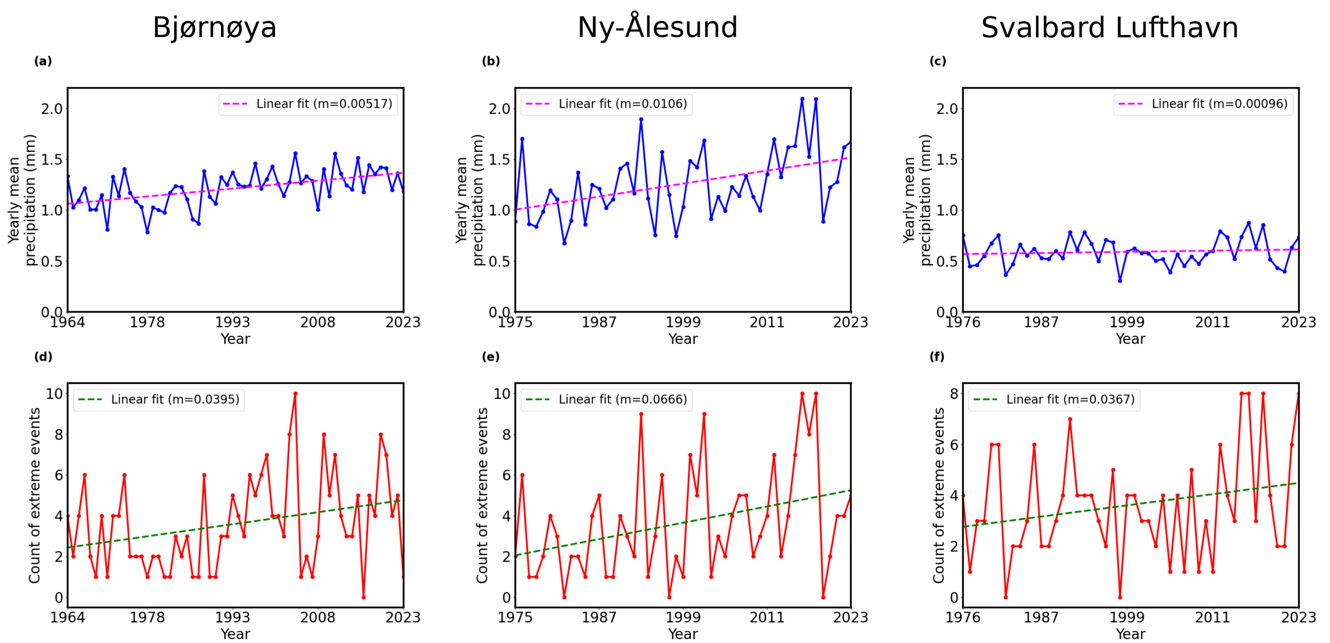

FIG. S1: (a-c) The variation of annual mean precipitation from Bjørnøya, Ny-Ålesund and Svalbard Lufthavn exhibits a linear trend fitted with a straight line (magenta dashed line). (d-f) The variation of number of extreme precipitation events exhibits a linear trend fitted with a straight line (green dashed line).

VII POT analysis of climate shifts based on temperature trends at each station

Based on the identified year of shift in surface air temperature trends at Bjørnøya, Ny-Ålesund, and Svalbard Lufthavn, the entire time period is divided into two distinct periods for each station. The shifts in temperature trends occurred in 1993 for Bjørnøya and 1999 for both Ny-Ålesund and Svalbard Lufthavn.

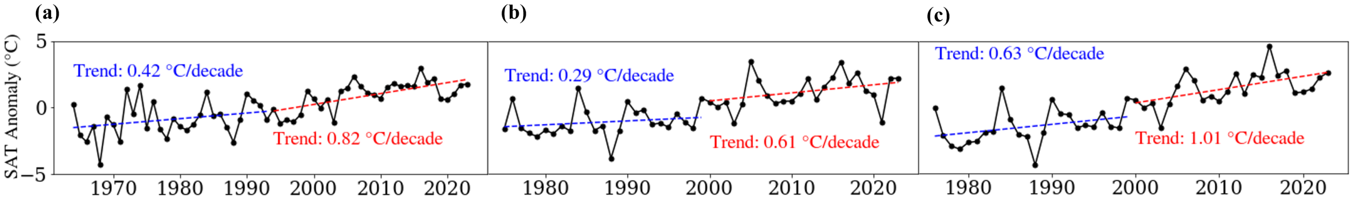

FIG. S2: Surface air temperature trends at (a) Bjørnøya, (b) Ny-Ålesund, and (c) Svalbard Lufthavn. The shift in trends began at different times for the three stations: in Bjørnøya, the shift started in 1993, whereas in Svalbard Lufthavn and Ny-Ålesund, the shift happened in 1999.

	Bjørnøya (1964-1992, 1993-2023)		Ny-Ålesund(1975-1998, 1999-2023)		Svalbard Lufthavn (1976-1998, 1999-2023)	
Time period	Shape (ξ)	100-year RL	Shape (ξ)	100-year RL	Shape (ξ)	100-year RL
1964-1992	0.13189	42.054	-	-	-	-
1993-2023	0.25598	62.13926	-	-	-	-
1975-1998	-	-	-0.09592	63.292	-	-
1999-2023	-	-	0.31325	134.5699	-	-
1976-1998	-	-	-	-	0.12716	39.590453
1999-2023	-	-	-	-	0.10045	40.3355

TABLE S1: Shape parameters and 100-year return levels (RL) for Bjørnøya, Ny-Ålesund, and Svalbard Lufthavn for their respective time periods.

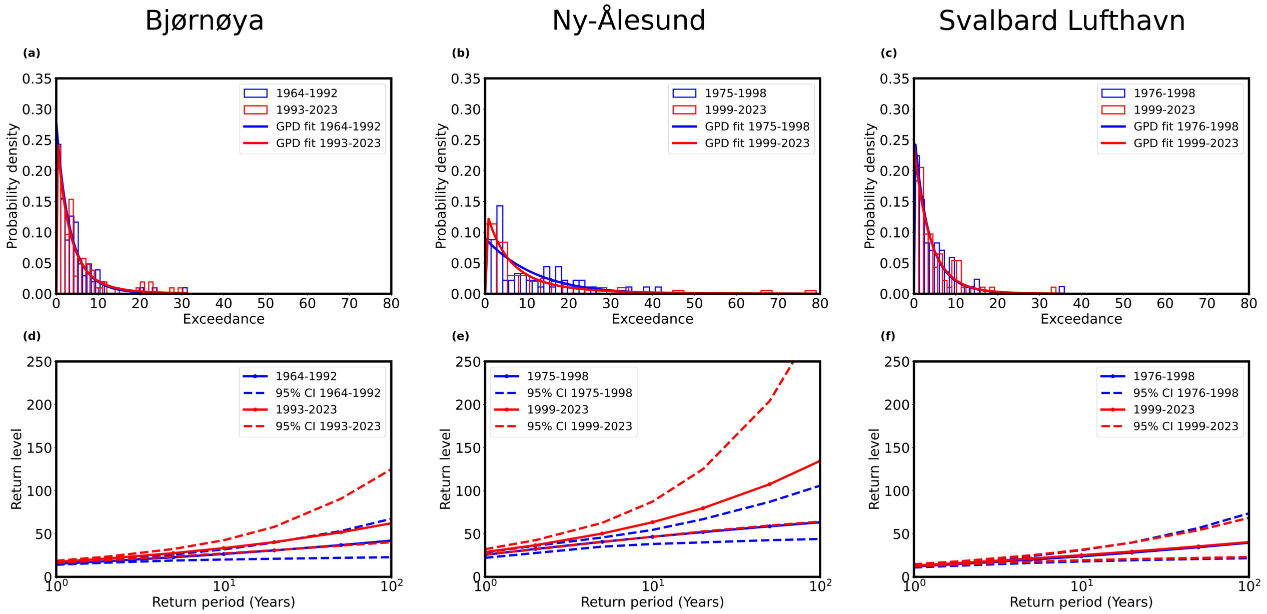

FIG. S3: (a-c) For two successive time periods, exceedance histograms and the estimated probability density function of the generalized Pareto distribution are displayed. (d-f) For each return period, we plot the return level (solid lines) with 95% confidence intervals (dashed lines). All of the plots are displayed in blue during the first time period, whereas all of the results are displayed in red during the second.

[S1] Stuart Coles, Joanna Bawa, Lesley Trenner, and Pat Dorazio. *An introduction to statistical modeling of extreme values*, volume 208. Springer, 2001.

[S2] Milan Gocic and Slavisa Trajkovic. Analysis of changes in meteorological variables using Mann-Kendall and Sen's slope estimator statistical tests in Serbia. *Global and Planetary Change*, 100:172–182, 2013.

[S3] Gottfried E Noether. Note on the Kolmogorov statistic in the discrete case. *Metrika*, 7(1):115–116, 1963.

[S4] Christoph Schär, Nikolina Ban, Erich M Fischer, Jan Rajczak, Jürg Schmidli, Christoph Frei, Filippo Giorgi, Thomas R Karl, Elizabeth J Kendon, Albert MG Klein Tank, et al. Percentile indices for assessing changes in heavy precipitation events. *Climatic Change*, 137:201–216, 2016.
